# Sleep patterns during infancy and neurodevelopmental and behavioral outcomes in early childhood: A prospective cohort study

**DOI:** 10.1002/jcv2.70155

**Published:** 2026-07-31

**Authors:** Wei Wei, Jun Zhang, Hui Wang

**Affiliations:** ^1^ Ministry of Education‐Shanghai Key Laboratory of Children's Environmental Health Xinhua Hospital Shanghai Jiao Tong University School of Medicine Shanghai China; ^2^ Ministry of Education‐Shanghai Key Laboratory of Children's Environmental Health, School of Public Health Shanghai Jiao Tong University School of Medicine Shanghai China

**Keywords:** birth cohort, infant sleep, neurodevelopmental and behavioral outcomes

## Abstract

**Background:**

Infant sleep is multidimensional, with sleep habits and sleep problems often co‐occurring and changing rapidly during early life. Evidence links infant sleep with later neurodevelopmental and behavioral outcomes, yet few studies have examined overall sleep patterns, their changes over time, and outcomes across multiple preschool domains. We examined associations of sleep patterns at 6 and 12 months and their transitions with preschool neurodevelopmental and behavioral outcomes.

**Methods:**

This prospective cohort study included 1707 children from the Shanghai Birth Cohort. Infant sleep at 6 and 12 months was assessed using the Brief Infant Sleep Questionnaire. Sleep patterns were identified at each time point using latent class analysis based on multiple sleep indicators, and children with sleep data at both ages were classified into transition groups. At age 4, outcomes were assessed using the Strengths and Difficulties Questionnaire (SDQ), Social Responsiveness Scale‐Short Form (SRS‐SF), and Behavior Rating Inventory of Executive Function‐Preschool Version (BRIEF‐P). Multivariable linear regression estimated associations.

**Results:**

At 6 and 12 months, 66.02% and 69.18% of infants were classified as having a good sleep pattern. Among children with sleep data at both time points, 51.57% had consistently good sleep, 16.68% improved, 14.44% deteriorated, and 17.31% had consistently poor sleep. Poor sleep at 6 months was associated with higher SDQ Total Difficulties scores. Poor sleep at 12 months was associated with higher SDQ Total Difficulties, SRS‐SF Total, and BRIEF‐P Global Executive Composite scores. Consistently poor sleep from 6 to 12 months was associated with less favorable outcomes than consistently good sleep.

**Conclusion:**

Poorer infant sleep patterns, especially at 12 months and when persistent from 6 to 12 months, were associated with less favorable preschool neurodevelopmental and behavioral outcomes. These findings support infant sleep patterns as an early‐life factor relevant to developmental research and monitoring.

## Key Points

**What's Known?**

- Infant sleep is multidimensional and may change substantially during early development, but its overall patterns and developmental changes remain insufficiently characterized.

**What's New?**

- In this prospective birth cohort, latent class analysis identified infant sleep patterns at 6 and 12 months and characterized transitions in sleep patterns from 6 to 12 months.
- Poor sleep at 12 months and consistently poor sleep from 6 to 12 months were associated with less favorable preschool neurodevelopmental and behavioral outcomes, including emotional and behavioral problems, social difficulties and autistic traits, and executive function.

**What's Relevant?**

- Infant sleep patterns may help inform developmental monitoring and support further research on early sleep as a modifiable factor related to child neurodevelopment.

## INTRODUCTION

Neurodevelopment involves multiple connected domains, including cognition, language, motor and sensory processing, social communication, emotional and behavioral regulation, and executive control (Ismail & Shapiro, 2019; Thapar et al., 2017). Developmental disabilities are common in childhood: in the United States, the proportion of 3‐ to 17‐year‐old children with diagnosed developmental disabilities increased from 16.2% in 2009–2011 to 17.8% in 2015–2017 (Zablotsky et al., 2019); a report from the United Nations Children's Fund and the World Health Organization placed the global estimate at approximately 317 million children and adolescents worldwide with health conditions linked to developmental disabilities in 2019 (World Health Organization & United Nations Children's Fund, 2023). Neurodevelopmental difficulties often begin early in life, frequently before children enter school, and may interfere with subsequent functioning across personal, social, academic, and later occupational domains (American Psychiatric Association, 2013). Their impact extends beyond the burden of diagnosed disorders and may affect children's learning, peer relationships, social participation, and later psychological health and overall well‐being (Black et al., 2017). Therefore, identifying early‐life factors associated with preschool neurodevelopmental and behavioral outcomes is important for preventing later developmental difficulties and promoting child health.

Sleep is a key modifiable behavior during early childhood. Infancy is a period of rapid sleep development, during which sleep‐wake rhythms, nighttime sleep consolidation, and sleep‐related behaviors undergo substantial changes (Bruni et al., 2014). Sleep is important for brain maturation, synaptic plasticity, circadian regulation, and the development of neural systems involved in emotional regulation, attention, inhibitory control, and social information processing (Cheng et al., 2021; Mäkelä et al., 2021; Raven et al., 2018). Therefore, sleep during this sensitive developmental period may be related to later differences across multiple neurodevelopmental domains.

Previous longitudinal studies have linked infant or toddler sleep with later neurodevelopmental and behavioral outcomes across several domains. For emotional and behavioral problems (EBPs), short sleep duration, frequent night wakings, and persistent severe sleep problems in infancy or toddlerhood have been associated with later EBPs (Cook et al., 2020; Sivertsen et al., 2015; Williamson et al., 2020). For social functioning and autistic traits, early sleep problems and infant sleep quality have been associated with later autism spectrum disorder (ASD) related outcomes (Kikuchi et al., 2023; Verhoeff et al., 2018). For executive function (EF), the proportion of sleep occurring during nighttime or daytime has been associated with later inhibitory control and working memory (Bernier et al., 2013; Morales‐Muñoz et al., 2021). However, prior research has mainly examined on specific sleep problems or single sleep dimensions.

Infant sleep is multidimensional, and sleep habit indicators, such as sleep initiation method, sleep location, and sleep position, may co‐occur with sleep quality indicators, such as sleep duration, night wakings, and snoring. Examining individual sleep variables may not fully capture the overall sleep profile during infancy. A pattern‐based approach can integrate multiple sleep indicators to better characterize infant sleep. As circadian rhythms and sleep organization become increasingly consolidated during infancy, sleep patterns may differ between 6 and 12 months. Characterizing both sleep patterns at specific infancy time points and their changes over time may help clarify whether persistent or changing sleep patterns are associated with later neurodevelopmental and behavioral outcomes.

Using data from a prospective birth cohort, we aimed to identify sleep patterns at 6 and 12 months of age and characterize changes in sleep patterns from 6 to 12 months. We further examined the associations of these sleep patterns and their changes with preschool neurodevelopmental and behavioral outcomes, including EBPs, social responsiveness and autistic traits, and EF.

## MATERIALS AND METHODS

### Study population

This study used data from participants in the Shanghai Birth Cohort (SBC), a cohort established to examine how genetic, behavioral, and environmental factors affect child growth, development, and disease risk (Zhang et al., 2019). Between 2013 and 2016, 4127 pregnant women were enrolled at six hospitals in Shanghai, resulting in 3692 singleton live births, with ongoing regular follow‐up assessments. In this study, children were included in each analysis if they had complete sleep data at the corresponding time point and complete data for at least one neurodevelopmental or behavioral outcome measure at age 4. Complete sleep data were defined as having all sleep variables used to identify sleep patterns available at that time point. Analyses of 6‐ and 12‐month sleep patterns included children with complete sleep data at the corresponding time point (*n* = 1342 and 1483, respectively). Children with complete sleep data at 6 months were included in the 6‐month sleep pattern analysis, regardless of whether they had complete sleep data at 12 months. Similarly, children with complete sleep data at 12 months were included in the 12‐month sleep pattern analysis, regardless of whether they had complete sleep data at 6 months. Only the sleep pattern transition analysis required complete sleep data at both 6 and 12 months (*n* = 1115) (Supporting Information S1: Figure S1). Written informed consent was provided by the caregivers of the children during each data collection. Ethical approval was granted by the ethics committees of the participating research institutions and hospitals, and the study was reported according to the STROBE guidelines. Data analyses were conducted from March 1 to November 30, 2024.

### Sleep assessment during infancy

Infant sleep was assessed at both 6 and 12 months using the Brief Infant Sleep Questionnaire (BISQ), which was completed by the parents. The BISQ is a multidimensional sleep assessment tool designed for children aged 0–3 years and has been widely used in research and clinical settings, including among Chinese populations (Mindell et al., 2010). It collects information on various sleep‐related factors, including sleep initiation methods, sleep location, preferred body position, bedtime, settling time, sleep duration, snoring, parent‐reported sleep difficulties, night wakings, and sleep regularity (Supporting Information S1: Table S1). These data were used to identify sleep patterns.

### Assessment of child neurodevelopmental and behavioral outcomes in early childhood

At age 4, child neurodevelopmental and behavioral outcomes were assessed using the parent‐reported Strengths and Difficulties Questionnaire (SDQ), the Social Responsiveness Scale‐Short Form (SRS‐SF), and the Behavior Rating Inventory of Executive Function‐Preschool Version (BRIEF‐P), which capture EBPs, social responsiveness and autistic traits, and EF, respectively.

The SDQ contains 25 items rated from 0 to 2 and covers five subscales: Emotional Symptoms, Conduct Problems, Hyperactivity, Peer Relationship Problems, and Prosocial Behavior. The Total Difficulties score is obtained by adding the first four subscales and ranges from 0 to 40, with larger values representing greater overall difficulties. Prosocial Behavior is scored separately on a 0–10 scale, with higher values reflecting better prosocial behavior (Goodman, 1997).

The SRS‐SF consists of 18 items, each scored from 0 to 3 and grouped into three factors: Social Cognition, Social Communication, and Autistic Mannerisms. All item scores were summed to obtain the total score (range: 0–54), higher values reflect more severe social deficits or autistic traits (Sturm et al., 2017).

The 63‐item BRIEF‐P uses a 3‐point response scale and comprises five subscales: Inhibit, Shift, Emotional Control, Working Memory, and Plan/Organize. These subscales are further grouped into three domains: Inhibitory Self‐Control Index, Flexibility Index (FI), and Emergent Metacognition Index. The Global Executive Composite (GEC) is calculated by summing the five clinical subscales, with a raw score range of 63–189. Following the BRIEF‐P manual, raw scores were converted to age‐ and sex‐standardized *T*‐scores; higher values denote poorer EF (Sherman & Brooks, 2010).

A brief description of what each subscale, index, and composite score assesses is provided in Supporting Information S1: Table S2. All three questionnaires have demonstrated acceptable reliability and validity among Chinese children (Chen et al., 2019; Du et al., 2008; Lu et al., 2017). The internal consistency of the SDQ, SRS‐SF, and BRIEF‐P scores in this sample was assessed using Cronbach's *α* and is shown in Supporting Information S1: Table S3. The Cronbach's *α* values for the composite scores were 0.72 for the SDQ Total Difficulties, 0.70 for the SRS‐SF Total, and 0.94 for the BRIEF‐P GEC.

### Covariates

Informed by prior literature and directed acyclic graphs (Supporting Information S1: Figure S2), covariates were selected because they may be associated with both infant sleep and preschool neurodevelopmental and behavioral outcomes. The final model included child characteristics, maternal and family sociodemographic factors, and postnatal caregiving factors. Specifically, covariates included child sex, child age at outcome assessment, maternal ethnicity, maternal age at delivery, parity, education, prenatal anxiety, self‐reported family economic status, breastfeeding practices, primary caregiver education, and recruitment hospital. Recruitment hospital was included to account for differences in recruitment across sites. These covariates were obtained from questionnaires or medical records during follow‐ups. Details on these measures are provided in Supporting Information S1: Appendix S1.

### Statistical analysis

Demographic characteristics and infant sleep dimensions were summarized using descriptive statistics. For descriptive analyses, continuous data were summarized as mean (SD), and categorical data were summarized as count (%).

We used latent class analysis (LCA) to construct infant sleep patterns based nine sleep variables from the BISQ, guided by pediatric sleep recommendations (China, 2017; Subspecialty Group of Pediatric Sleep, 2023): sleep initiation methods, sleep location, preferred body position, bedtime, settling time, total sleep duration, snoring, parent‐reported sleep difficulties, and night wakings. Sleep regularity was not included in the LCA model because 97% of children were reported to have regular sleep. For inclusion in the LCA models, these variables were recoded into binary indicators according to pediatric sleep recommendations, including independent sleep initiation, room‐sharing with parents, back sleeping, bedtime at or before 9:00 p.m., settling time <20 min, age‐appropriate total sleep duration, no snoring, no parent‐reported sleep difficulty, and night wakings <2. The original categories and corresponding binary indicators used in the LCA models are presented in Supporting Information S1: Table S1.

LCA was conducted separately for sleep variables measured at 6 and 12 months using the “poLCA” package in R (Linzer and Lewis, 2011). LCA is an appropriate method for analyzing categorical variables, enabling the identification of latent subgroups based on multiple observed characteristics (Sinha et al., 2021; Weller et al., 2020).

LCA was used to identify latent sleep patterns based on infants' response profiles across the nine sleep indicators from the BISQ at each time point. Models with one to six latent classes were fitted separately at 6 and 12 months. The number of classes was selected mainly according to the Bayesian Information Criterion, together with the Akaike Information Criterion, likelihood ratio statistic (*G* ^2^), Pearson's chi‐square statistic (*χ* ^2^), entropy, class size, and interpretability. Participants were classified into the latent class for which they had the highest posterior probability. Latent classes were interpreted and named using the conditional item‐response probabilities of the sleep indicators. To examine changes in sleep patterns from 6 to 12 months, participants with sleep pattern classifications at both time points were further categorized by cross‐tabulating their sleep patterns at 6 and 12 months.

Multivariable linear regression was applied to assess associations of 6‐ and 12‐month sleep patterns, as well as pattern transitions, with preschool neurodevelopmental and behavioral outcomes. Missing values of covariates were addressed using multiple imputation with 10 imputed datasets. Sleep exposures and neurodevelopmental and behavioral outcomes were not imputed; however, they were included as predictors in the imputation model for incomplete covariates. Estimates from the imputed datasets were pooled using Rubin's rules (van Buuren, 2018; van Buuren & Groothuis‐Oudshoorn, 2011).

Several sensitivity analyses were performed to assess the stability of the results. First, because preterm birth is an important perinatal factor that may be related to both infant sleep and later child neurodevelopmental and behavioral outcomes, we repeated the main analyses with additional adjustment for preterm birth (yes/no). Second, to account for the potential impact of postnatal adversity, we repeated the analyses with additional adjustment for the Child Stress Disorders Checklist total score and the occurrence of accidents (yes/no), as outlined in Supporting Information S1: Appendix S1.

Statistical significance was defined as *p* < .05. Analyses were conducted in R version 4.4.1.

## RESULTS

### Characteristics of the study population

Among the 1707 participants, 1342 had sleep data at 6 months and 1483 had sleep data at 12 months, 1115 had data available for sleep pattern transitions. Among the participants, 52.43% were boys, and over 98% were of Han ethnicity. The majority of mothers were primiparous (85.98%) and held a bachelor's degree or higher (64.94%). Additionally, 88.49% of the participants reported moderate or adequate family economic status (Table 1). Included and excluded children showed no significant demographic differences (Supporting Information S1: Table S4).

**TABLE 1:** Demographic characteristics of participants included in the analyses (*N* = 1707).

Characteristic	Mean (SD) or *N* (%)
Child sex	
Boys	895 (52.43)
Girls	812 (47.57)
Missing	0
Child age, mean (SD), years	4.50 (0.27)
Missing	0
Breastfeeding status at 6 months	
Not breastfed	81 (5.15)
Breastfed	1491 (94.85)
Missing	135
Breastfeeding status at 12 months	
Never breastfed	43 (2.60)
Stopped breastfeeding	1091 (66.08)
Still breastfeeding	517 (31.31)
Missing	56
Primary caregiver's education at 6 months	
Below high school	486 (31.91)
High school	407 (26.72)
Above high school	630 (41.37)
Missing	184
Primary caregiver's education at 12 months	
Below high school	698 (44.71)
High school	485 (31.07)
Above high school	378 (24.22)
Missing	146
Maternal ethnicity	
Han	1631 (98.79)
Non‐Han	20 (1.21)
Missing	56
Maternal age at delivery, mean (SD), years	28.76 (3.70)
Missing	58
Parity	
0	1417 (85.98)
≥1	231 (14.02)
Missing	59
Premature birth	
Yes	78 (4.71)
No	1577 (95.29)
Missing	52
Maternal prenatal anxiety	
Normal	1474 (93.89)
Minimal	86 (5.48)
Moderate	10 (0.64)
Missing	137
Maternal education	
Less than bachelor's degree	579 (35.07)
Bachelor's degree	870 (52.70)
Graduate degree	202 (12.24)
Missing	56
Self‐reported family economic status	
Adequate	298 (18.53)
Moderate	1125 (69.96)
Tight	185 (11.50)
Missing	99
SDQ—Total difficulties score	9.15 (4.33)
Missing	4
SRS‐SF—Total score	7.20 (4.10)
Missing	13
BRIEF‐P—Global Executive Composite score	47.27 (8.69)
Missing	38

Abbreviations: BRIEF‐P, Behavior Rating Inventory of Executive Function‐Preschool Version; SDQ, Strengths and Difficulties Questionnaire; SRS‐SF, Social Responsiveness Scale‐Short Form.

### Infant sleep patterns

**FIGURE 1:**
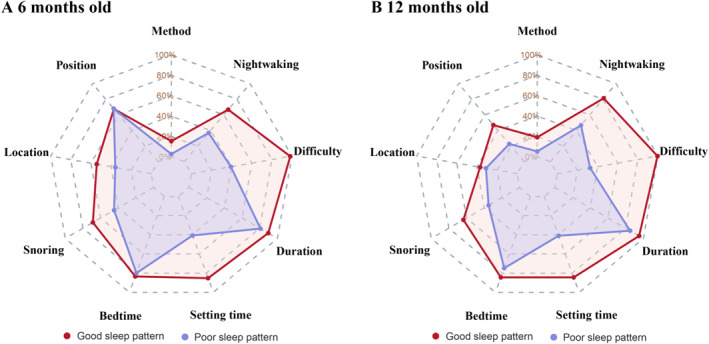
Latent class analysis of infant sleep patterns at 6 and 12 months. Radar charts show the estimated class‐conditional probabilities for each sleep indicator in the good and poor sleep pattern groups at 6 months (A) and 12 months (B). Higher values indicate a higher probability of the category coded as 1 in the latent class analysis.

Supporting Information S1: Table S5 presents the characteristics of infant sleep variables. Based on model fit indices (Supporting Information S1: Tables S6 and S7) and interpretability, a 2‐class model was selected at both time points (Figure 1). According to the conditional item‐response probabilities, one class at each time point was characterized by higher probabilities of favorable sleep habits and fewer sleep problems and was labeled as the “good sleep pattern.” The other class was characterized by less favorable sleep habits and more sleep problems and was labeled as the “poor sleep pattern.” At 6 and 12 months, 66.02% and 69.18% of children were classified into the good sleep pattern group, respectively.

Based on the sleep patterns at 6 and 12 months, children were further categorized into four transition groups: consistently good, improved, deteriorated, and consistently poor. Consistently good was defined as a good sleep pattern at both 6 and 12 months; improved as a poor sleep pattern at 6 months followed by a good sleep pattern at 12 months; deteriorated as a good sleep pattern at 6 months followed by a poor sleep pattern at 12 months; and consistently poor as a poor sleep pattern at both time points. The proportions of these groups were 51.57%, 16.68%, 14.44%, and 17.31%, respectively (Supporting Information S1: Figure S3).

### Associations between infant sleep patterns and preschool neurodevelopmental and behavioral outcomes

**FIGURE 2:**
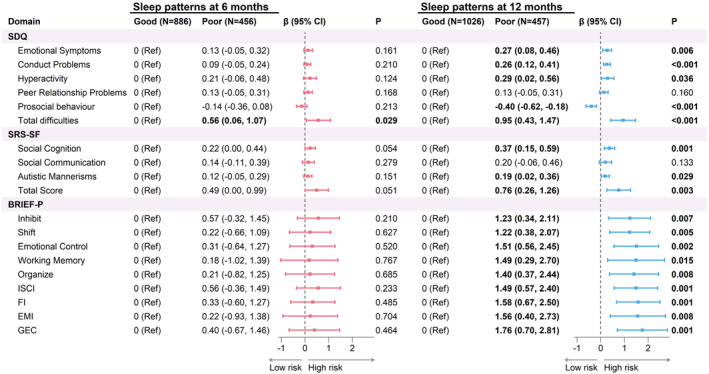
Associations between sleep patterns at 6 and 12 months and preschool neurodevelopmental and behavioral outcomes Adjusted for child sex, child age, maternal ethnicity, maternal age at delivery, parity, maternal education, maternal prenatal anxiety, self‐reported family economic status, recruitment hospital, breastfeeding status at 6/12 months, and primary caregiver's education at 6/12 months. The BRIEF‐P did not adjust for child sex and age because we used standardized *T*‐scores. *β*, coefficients; BRIEF‐P, Behavior Rating Inventory of Executive Function–Preschool Version; CI, confidence interval; EMI, Emergent Metacognition Index; FI, Flexibility Index; GEC, Global Executive Composite; ISCI, Inhibitory Self‐Control Index; SDQ, Strengths and Difficulties Questionnaire; SRS‐SF, Social Responsiveness Scale–Short Form.

Compared with children with a good sleep pattern at 6 months, those with a poor sleep pattern had higher scores on the SDQ Total Difficulties scale at age 4 (*β* = .56, 95% CI: 0.06, 1.07), while no significant associations were observed for the other SDQ, SRS‐SF, or BRIEF‐P outcomes. Compared with children with a good sleep pattern at 12 months, those with a poor sleep pattern had higher scores on the SDQ Total Difficulties scale (*β* = .95, 95% CI: 0.43, 1.47), the SRS‐SF total score (*β* = .76, 95% CI: 0.26, 1.26), and the BRIEF‐P GEC (*β* = 1.76, 95% CI: 0.70, 2.81), indicating more EBPs, poorer social responsiveness and more autistic traits, and poorer EF, respectively. The associations between sleep patterns and preschool neurodevelopmental and behavioral outcomes were stronger at 12 months than at 6 months (Figure 2).

### Associations between changes in infant sleep patterns and preschool neurodevelopmental and behavioral outcomes

Compared with children with consistently good sleep patterns from 6 to 12 months, children in the improved group showed no significant differences in SDQ, SRS‐SF, or BRIEF‐P outcomes. The deteriorated group showed lower SDQ Prosocial Behavior scores (*β* = −.44, 95% CI: −0.78, −0.11) and higher BRIEF‐P Emotional Control, Organize, and FI scores. Children with consistently poor sleep patterns had higher scores on the SDQ Total Difficulties scale (*β* = 1.19, 95% CI: 0.43, 1.95), the SRS‐SF total score (*β* = 1.20, 95% CI: 0.46, 1.93), and the BRIEF‐P GEC (*β* = 2.15, 95% CI: 0.60, 3.71). The consistently poor group also had lower SDQ Prosocial Behavior scores (*β* = −.57, 95% CI: −0.89, −0.25). Significant trends across sleep pattern transition groups suggested that less favorable sleep pattern changes from 6 to 12 months were associated with poorer preschool neurodevelopmental and behavioral outcomes (Table 2).

**TABLE 2:** Associations between changes in infant sleep patterns from 6 to 12 months and preschool neurodevelopmental and behavioral outcomes (*N* = 1115).

	Consistently good (*N* = 575)	Improved (*N* = 186)	Deteriorated (*N* = 161)	Consistently poor (*N* = 193)	*p* for trend
		*β* (95% CI)	*β* (95% CI)	*β* (95% CI)	
SDQ					
Emotional symptoms	0 (ref)	.17 (−0.10, 0.44)	.15 (−0.14, 0.44)	**.29 (0.01, 0.57)**	**.046**
Conduct problems	0 (ref)	−.02 (−0.23, 0.18)	.16 (−0.06, 0.38)	**.24 (0.03, 0.45)**	**.014**
Hyperactivity	0 (ref)	.25 (−0.14, 0.64)	.21 (−0.21, 0.63)	.39 (−0.01, 0.79)	.065
Peer relationship problems	0 (ref)	.09 (−0.18, 0.35)	.07 (−0.21, 0.36)	.27 (−0.01, 0.54)	.076
Prosocial behavior	0 (ref)	−.20 (−0.51, 0.12)	**−.44 (−0.78, −0.11)**	**−.57 (−0.89, −0.25)**	**<.001**
Total difficulties	0 (ref)	.49 (−0.25, 1.22)	.59 (−0.20, 1.39)	**1.19 (0.43, 1.95)**	**.002**
SRS‐SF					
Social cognition	0 (ref)	.18 (−0.14, 0.50)	.27 (−0.07, 0.62)	**.55 (0.22, 0.88)**	**.001**
Social communication	0 (ref)	.16 (−0.21, 0.53)	.02 (−0.38, 0.41)	.32 (−0.06, 0.70)	.167
Autistic Mannerisms	0 (ref)	.12 (−0.12, 0.36)	.02 (−0.24, 0.28)	**.32 (0.07, 0.57)**	**.033**
Total score	0 (ref)	.48 (−0.24, 1.19)	.31 (−0.46, 1.08)	**1.20 (0.46, 1.93)**	**.004**
BRIEF‐P					
Inhibit	0 (ref)	.67 (−0.59, 1.94)	.83 (−0.53, 2.19)	**2.02 (0.72, 3.31)**	**.003**
Shift	0 (ref)	.17 (−1.06, 1.40)	1.14 (−0.19, 2.46)	**1.39 (0.12, 2.65)**	**.016**
Emotional control	0 (ref)	.41 (−0.93, 1.75)	**1.55 (0.10, 3.00)**	**1.76 (0.38, 3.14)**	**.005**
Working memory	0 (ref)	.08 (−1.66, 1.83)	1.20 (−0.68, 3.08)	1.65 (−0.14, 3.44)	**.046**
Organize	0 (ref)	.31 (−1.17, 1.80)	**1.90 (0.30, 3.49)**	1.42 (−0.10, 2.94)	**.020**
ISCI	0 (ref)	.67 (−0.63, 1.96)	1.22 (−0.18, 2.62)	**2.15 (0.82, 3.49)**	**.001**
FI	0 (ref)	.37 (−0.94, 1.67)	**1.56 (0.15, 2.96)**	**1.80 (0.46, 3.14)**	**.003**
EMI	0 (ref)	.18 (−1.50, 1.85)	1.57 (−0.24, 3.37)	1.70 (−0.02, 3.42)	**.025**
GEC	0 (ref)	.46 (−1.06, 1.97)	1.62 (−0.01, 3.25)	**2.15 (0.60, 3.71)**	**.003**

*Note*: Adjusted for child sex, child age, maternal ethnicity, maternal age at delivery, parity, maternal education, maternal prenatal anxiety, self‐reported family economic status, recruitment hospital, breastfeeding status at 6 months, and primary caregiver's education at 6 and 12 months. The BRIEF‐P did not adjust for child sex and age because we used standardized *T*‐scores. Bold values indicate statistically significant estimates or *p* values <.05.

Abbreviations: *β*, coefficients; BRIEF‐P, Behavior Rating Inventory of Executive Function–Preschool Version; CI, confidence interval; EMI, Emergent Metacognition Index; FI, Flexibility Index; GEC, Global Executive Composite; ISCI, Inhibitory Self‐Control Index; SDQ, Strengths and Difficulties Questionnaire; SRS‐SF, Social Responsiveness Scale–Short Form.

### Sensitivity analyses

Sensitivity analyses showed that the results were generally consistent with the main analyses. Additional adjustment for preterm birth, the Child Stress Disorders Checklist total score, or accident occurrence did not change the associations of sleep patterns at 6 months, sleep patterns at 12 months, or changes in sleep patterns from 6 to 12 months with preschool neurodevelopmental and behavioral outcomes (Supporting Information S1: Tables S8–S13).

## DISCUSSION

In this prospective birth cohort study, poorer sleep patterns during infancy were associated with less favorable preschool neurodevelopmental and behavioral outcomes, including more EBPs, poorer social responsiveness and more autistic traits, and poorer EF. Children with consistently poor sleep patterns from 6 to 12 months showed less favorable outcomes across these domains than those with consistently good sleep patterns.

Our findings for the SDQ outcomes are consistent with longitudinal evidence linking early sleep problems with later EBPs (Fulfs et al., 2024). In a large Norwegian cohort, short sleep duration and frequent night awakenings at 18 months were linked to internalizing and externalizing problems at 5 years, even after adjustment for EBPs at 18 months (Sivertsen et al., 2015). Cook et al. also reported that persistent severe sleep problems in the first year of life were associated with an increased risk of emotional symptoms at age 4 and emotional disorders at age 10 (Cook et al., 2020). Evidence from the Australian Children also showed that persistent sleep‐problem trajectories spanning infancy to middle childhood were linked to higher EBPs at ages 10–11 years (Williamson et al., 2020). Compared with these previous studies, our study used a pattern‐based approach to capture the co‐occurrence of multiple sleep habit and sleep quality indicators during infancy.

Evidence linking early sleep with later social functioning and autistic traits remains relatively limited. In a Japanese longitudinal study, sleep onset before 22:00 and longer total or nighttime sleep at 18 months were linked to more favorable social competence trajectories from 18 to 42 months, suggesting that early sleep timing and duration may be related to subsequent social development (Tomisaki et al., 2018). For autistic traits, previous research in older children suggests that sleep problems commonly co‐occur with ASD‐related problems. In a population‐based longitudinal study of school‐aged children, Sivertsen et al. (2012) reported that children with autism spectrum problems had a higher prevalence and greater chronicity of insomnia than controls, and that autism spectrum problems predicted later sleep problems. Evidence from a younger population‐based cohort has further examined the temporal relationship between sleep problems and autistic traits. In the Generation R cohort, sleep problems were assessed repeatedly from 1.5 to 9 years, while autistic traits were assessed at 1.5, 3, and 6 years. Early‐childhood sleep problems were linked to higher subsequent SRS scores, but the association was no longer evident after baseline autistic traits were controlled for. Conversely, autistic traits and ASD diagnosis predicted later sleep problems even after adjustment for baseline sleep problems (Verhoeff et al., 2018). These findings suggest that sleep problems may occur alongside early autistic traits rather than simply precede them. In our study, poor sleep patterns during infancy were associated with higher SRS‐SF scores at age 4. However, because we did not adjust for baseline social responsiveness or autistic traits, we could not determine whether a poor infant sleep pattern preceded later social difficulties or partly reflected pre‐existing social developmental differences.

Prior evidence has linked infant sleep to later EF. Bernier et al. (2013) reported that a greater proportion of nighttime sleep at 1 year was linked to better task‐based complex EF at 4 years, measured using the Wechsler Preschool and Primary Scale of Intelligence. Morales‐Muñoz et al. (2021) assessed task‐based inhibitory control and working memory at 30 months and reported that daytime sleep proportion at 12 months was associated with inhibitory control, whereas longer nighttime wakefulness was associated with poorer working memory. Evidence from ASD populations also suggests that early sleep may be related to later EF development. Tesfaye et al. (2021) found that sleep onset before age 5 was associated with later trajectories of teacher‐reported behavioral regulation, although not with metacognition. Our study adds to this literature by examining overall infant sleep patterns in a birth cohort and using the BRIEF‐P to assess multiple domains of everyday EF in preschool children.

Infant sleep is composed of multiple interrelated sleep habit and sleep quality indicators (Grandner & Fernandez, 2021; Meltzer et al., 2021). However, the relative importance of individual sleep indicators remains unclear and may depend on infant age, caregiving practices, and cultural context. These indicators are not isolated from one another, but may jointly reflect sleep organization, sleep consolidation, and the caregiving context. For example, bedtime, sleep initiation method, sleep duration, and night wakings may together reflect the stability and continuity of sleep, whereas sleep location and sleep position may be more strongly influenced by caregiving practices and cultural habits (Kruse et al., 2024). Based on this multidimensional nature of infant sleep, we integrated multiple sleep indicators and used LCA to identify overall sleep patterns at 6 and 12 months, and further examined changes in sleep patterns from 6 to 12 months. Compared with previous studies that mainly focused on individual sleep dimensions, this pattern‐based approach may better capture the real‐world co‐occurrence of infant sleep behaviors and sleep problems, and offer a broader description of the early sleep context in relation to later neurodevelopmental and behavioral outcomes.

Several neurobiological pathways may underlie the associations observed in this study. During infancy and early childhood, sleep supports synaptic plasticity, memory consolidation, and neural maturation, processes that are important for later neurodevelopmental functioning (Diekelmann & Born, 2010; Tononi & Cirelli, 2014). Poor sleep quality or fragmented sleep may affect emotional regulation through fronto‐limbic circuitry. Neuroimaging evidence suggests that sleep deprivation may heighten amygdala reactivity to negative emotional stimuli and attenuate amygdala–medial prefrontal functional coupling, a pathway supporting top‐down emotional regulation (Krause et al., 2017; Yoo et al., 2007). Such alterations may contribute to irritability, lower frustration tolerance, and difficulties in regulating negative emotions, which may partly explain the associations with higher SDQ scores. Sleep may also be related to social developmental outcomes through its effects on attention to and processing of social cues. Children's social development depends in part on their ability to orient to, sustain attention to, and interpret socially relevant cues, including eyes, faces, gaze, and facial emotional expressions. Social attention to these cues is important for social information processing and has been linked to autistic traits (Falck‐Ytter et al., 2023; Lin et al., 2020). Evidence also suggests that sleep loss can impair attentional control and facial emotion recognition, which may reduce children's ability to process and respond to social cues (Cohen et al., 2021; Krause et al., 2017; van der Helm et al., 2010). This pathway may partly explain the observed association with higher SRS‐SF scores. For EF, the prefrontal cortex and frontoparietal networks are central to inhibitory control, working memory, cognitive flexibility, and planning, and these systems may be particularly sensitive to sleep disruption during development (Diamond, 2020; Friedman & Robbins, 2022). In addition to these neurobiological pathways, sleep‐related behaviors such as difficulty initiating sleep independently and frequent night wakings may reflect early self‐regulatory difficulties or caregiving patterns, which could contribute to later neurodevelopmental and behavioral outcomes (Asmussen et al., 2024).

### Strengths and limitations

This study has some strengths. Firstly, the well‐designed birth cohort provided comprehensive information on potential confounders, enabling us to control these variables more precisely. For instance, breastfeeding status and the primary caregiver may vary across time points. By adjusting for these variables at both 6 and 12 months separately, we were able to control their time‐specific influences, thereby enhancing the validity of our findings. Furthermore, we used multiple validated instruments to assess preschool neurodevelopmental and behavioral outcomes. The consistency and complementarity of results across different scales enhanced the robustness and reliability of the findings.

Our study also has several limitations. Firstly, both infant sleep and child neurodevelopmental and behavioral outcomes were reported by caregivers, which may have introduced reporting bias. Caregivers may overestimate sleep duration, underestimate night awakenings, or rate children with more challenging behaviors more negatively across multiple domains (Alacha & Lefler, 2021; Najman et al., 2001; Quante et al., 2021; Sadeh, 1996). Future research should include objective sleep assessments, such as actigraphy, and use neurodevelopmental and behavioral assessments based on multiple informants, such as teacher reports and clinical evaluations. Secondly, we did not assess temperament or other early neurodevelopmental and behavioral characteristics during infancy. Therefore, we could not adjust for baseline child characteristics that may influence both infant sleep and later neurodevelopmental and behavioral outcomes. Residual confounding or potential reverse causality cannot be fully excluded. Future studies with repeated assessments of sleep, temperament, and neurodevelopmental and behavioral outcomes from infancy onward are needed to better clarify the directionality of these associations. Lastly, most participants had well‐educated mothers and favorable economic status, which may limit the applicability of our findings to less economically advantaged populations.

## CONCLUSION

This prospective birth cohort study found that poorer sleep patterns in infancy, particularly at 12 months, were associated with less favorable preschool neurodevelopmental and behavioral outcomes, including more EBPs, poorer social responsiveness and more autistic traits, and poorer EF. Children with consistently poor sleep patterns from 6 to 12 months showed poorer preschool neurodevelopmental and behavioral outcomes, whereas those with consistently good sleep patterns had the most favorable outcomes. These findings highlight the importance of considering infant sleep as a multidimensional pattern and suggest that early sleep patterns may provide useful information for identifying children who could benefit from closer developmental monitoring and for informing early‐life strategies to promote child health.

## AUTHOR CONTRIBUTIONS

**Wei Wei**: Statistical Analysis; formal analysis; data curation; writing—original draft preparation; writing—review and editing. **Jun Zhang**: Conceptualization; study design; formal analysis; data curation; writing—review and editing. **Hui Wang**: Conceptualization; study design; formal analysis; data curation; writing—review and editing.

## CONFLICT OF INTEREST STATEMENT

The authors declare no conflicts of interest.

## ETHICAL CONSIDERATIONS

The 6‐ and 12‐month infant follow‐up assessments were approved by the Ethics Committee of Xinhua Hospital Affiliated to Shanghai Jiao Tong University School of Medicine (approval number: XHEC‐C‐2013‐001; approval date: January 7, 2013). The 4‐year child follow‐up assessment was approved by the same ethics committee as an amendment to the original protocol (approval number: XHEC‐C‐2013‐001‐3; approval date: May 2, 2018). Written informed consent was obtained from the participating mothers/parents or legal guardians for cohort participation and follow‐up assessments. Child assent was not applicable because the children were infants or preschool‐aged at the time of assessment.

## Supporting information

Supporting Information S1

## Data Availability

The data that support the findings of this study are available on request from the corresponding author. The data are not publicly available due to privacy or ethical restrictions.
