# Supplementary material for: Sleep patterns during infancy and neurodevelopmental and behavioral outcomes in early childhood: A prospective cohort study
Source: JCPP Adv. 2026 Jul 31:e70155. Online ahead of print. doi: 10.1002/jcv2.70155 (PMC13428066; doi:10.1002/jcv2.70155)
Supplement: Supplementary file 1 — Supporting Information S1 [file JCV2-9999-e70155-s001.docx]

**Sleep patterns during infancy and neurodevelopmental and behavioral outcomes in early childhood: a prospective cohort study**

**Supporting information**

**Table of Contents**

[Appendix S1. Method: Measures of covariates 4](#_Toc233706685)

[Figure S1. Flow chart for participant selection 6](#_Toc233706686)

[Figure S2. Directed Acyclic Graph for covariate selection 7](#_Toc233706687)

[Figure S3. Sankey diagram of Infant Sleep Patterns Transition (N =1,115) 8](#_Toc233706688)

[Table S1. Assignment of Sleep Variables 9](#_Toc233706689)

[Table S2. Domains assessed by the Strengths and Difficulties Questionnaire (SDQ), the Social Responsiveness Scale-Short Form (SRS-SF), and the Behavior Rating Inventory of Executive Function-Preschool Version (BRIEF-P) 10](#_Toc233706690)

[Table S3. Internal Consistency of Child Neurodevelopmental and Behavioral Outcome Measures 11](#_Toc233706691)

[Table S4. Demographic Characteristics of Participants Included in and Excluded from Analyses 12](#_Toc233706692)

[Table S5. Infant Sleep Characteristics at 6 and 12 Months of Age (N=1,707) 14](#_Toc233706693)

[Table S6. Model fit indices and class distribution for the 6-month latent class analysis (LCA) models 16](#_Toc233706694)

[Table S7. Model fit indices and class distribution for the 12-month latent class analysis (LCA) models 17](#_Toc233706695)

[Table S8. Associations between Sleep Patterns at 6 and 12 Months and Preschool Neurodevelopmental and Behavioral Outcomes, with Additional Adjustment for Preterm Birth 18](#_Toc233706696)

[Table S9. Associations between Changes in Infant Sleep Patterns from 6 to 12 Months and Preschool Neurodevelopmental and Behavioral Outcomes, with Additional Adjustment for Preterm Birth (N = 1,115) 19](#_Toc233706697)

[Table S10. Associations between Sleep Patterns at 6 and 12 Months and Preschool Neurodevelopmental and Behavioral Outcomes, with Additional Adjustment for Stress Disorders 20](#_Toc233706698)

[Table S11. Associations between Changes in Infant Sleep Patterns from 6 to 12 Months and Preschool Neurodevelopmental and Behavioral Outcomes, with Additional Adjustment for Stress Disorders (N = 1,115) 21](#_Toc233706699)

[Table S12. Associations between Sleep Patterns at 6 and 12 Months and Preschool Neurodevelopmental and Behavioral Outcomes, with Additional Adjustment for Accident Occurrence 22](#_Toc233706700)

[Table S13. Associations between Changes in Infant Sleep Patterns from 6 to 12 Months and Preschool Neurodevelopmental and Behavioral Outcomes, with Additional Adjustment for Accident Occurrence (N = 1,115) 23](#_Toc233706701)

Appendix S1. Method: Measures of covariates

**Maternal characteristics**: Maternal ethnicity (Han/Non-Han), age at delivery (continuous), parity (0/≥1), and education level (below Bachelor's degree/Bachelor's degree/above Bachelor's degree) were obtained from questionnaires or medical records during follow-ups. Maternal prenatal anxiety was assessed in the second trimester using the Self-Rating Anxiety Scale (SAS), with standardized scores categorized as follows: <50 (no anxiety), 50–59 (mild anxiety), 60–69 (moderate anxiety), and ≥70 (severe anxiety).

**Self-reported family economic status**: During the second-trimester follow-up, participants were asked, "How would you describe your current financial situation?" Response options included "Living comfortably," "It's okay," "Budgeting carefully," "Struggling," and "In debt." Based on these responses, family economic status was classified into three categories: adequate ("Living comfortably"), moderate ("It's okay"), and tight ("Budgeting carefully," "Struggling," or "In debt").

**Recruitment hospitals**: Hospitals were categorized into four groups based on geographical location: (1) Xinhua Hospital or Fudan University Obstetrics and Gynecology Hospital, (2) Xinhua Hospital Chongming Branch, (3) International Peace Maternity and Child Hospital, and (4) Shanghai Children's Medical Center.

**Breastfeeding status**: At the 6-month follow-up, breastfeeding status was assessed with the question, "Is the baby currently being breastfed?" At the 12-month follow-up, participants were asked, "At what age did the child completely stop breastfeeding?" with response options: "Never breastfed," "Stopped breastfeeding," and "Still breastfeeding."

**Primary caregiver's education**: Collected during follow-up and categorized into three levels: below high school, high school, and above high school.

**Measures of Accident Occurrence:** A binary variable for accident occurrence (yes/no) was determined by parents' responses during the 4-year follow-up to the question: "Has your child experienced the following accidents since 24 months of age?"

**Measures of Stress Disorders:** Stress disorders were assessed through the traumatic event item of the Child Stress Disorders Checklist (CSDC)(Saxe et al., 2003), completed by parents during the 4-year follow-up. This section provides detailed information of traumatic events. Each event was scored as 0 (not experienced), 1 (witnessed), or 2 (personally experienced). A higher total score of all events indicates a more severe stress disorder in children.

**Reference**

SAXE, G., CHAWLA, N., STODDARD, F., KASSAM-ADAMS, N., COURTNEY, D., CUNNINGHAM, K., LOPEZ, C., HALL, E., SHERIDAN, R., KING, D. & KING, L. (2003). Child Stress Disorders Checklist: a measure of ASD and PTSD in children. *Journal of the American Academy of Child and Adolescent Psychiatry,* 42**,** 972-978.


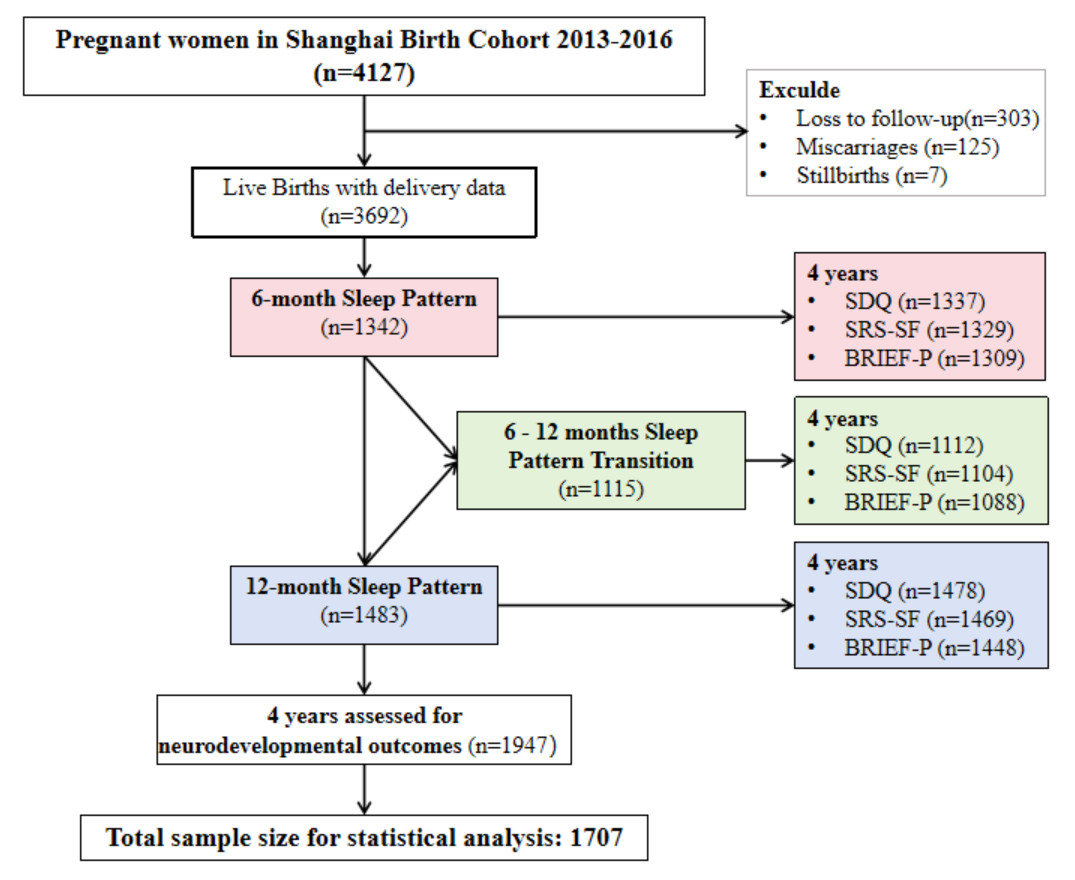


Figure S1. Flow chart for participant selection


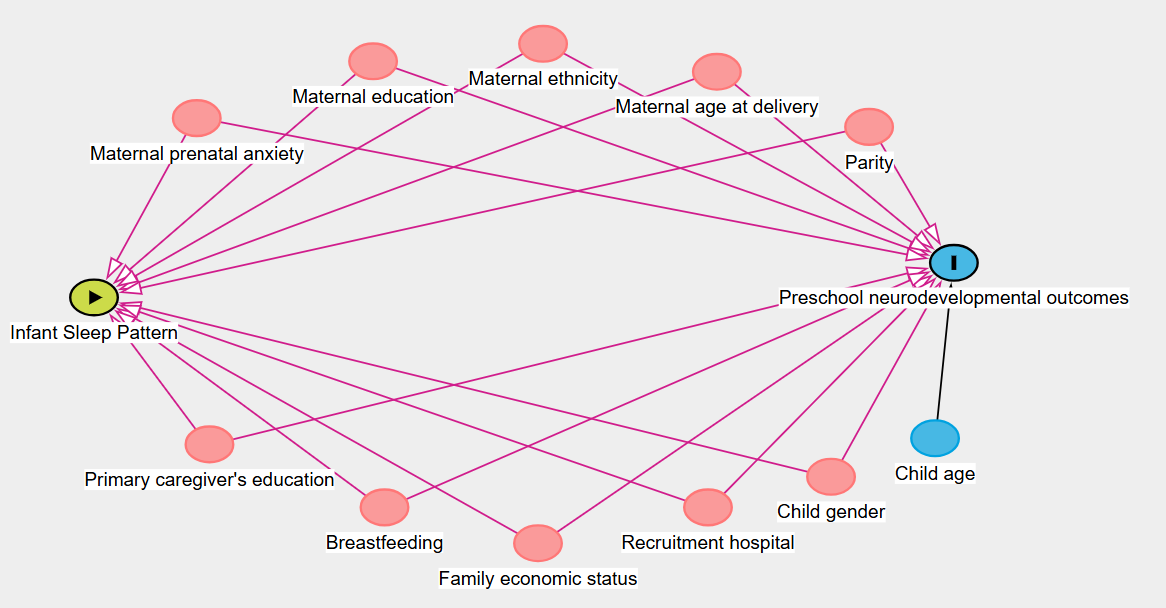


Figure S2. Directed Acyclic Graph for covariate selection


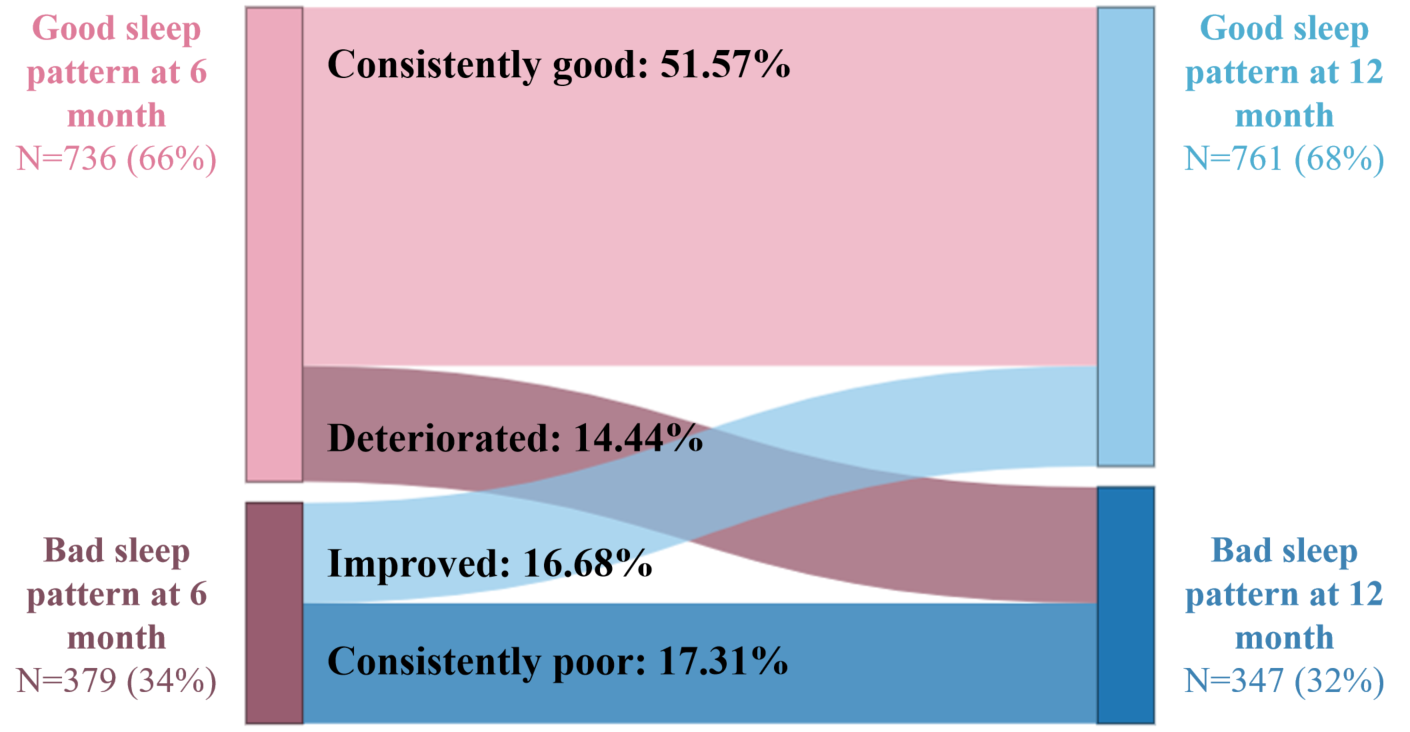


Figure S3. Sankey diagram of Infant Sleep Patterns Transition (N =1,115)

Table S1. Assignment of Sleep Variables

| Sleep Variable | Original Assignment | Recoded Assignment Included in LCA Model |
| --- | --- | --- |
| Sleep initiation methods | In bed alone = 1; Feeding to sleep = 2; Rocking to sleep = 3; Being held to sleep = 4; In bed near parent = 5 | In bed alone = 1; Other = 2 |
| Sleep location | Room-sharing with parents = 1; Bed-sharing with parents = 2; Crib in own room = 3; Room-sharing with sibling = 4 | Room-sharing with parents = 1; Other = 2 |
| Preferred body position | Back = 1; Side = 2; Stomach = 3 | Back = 1; Other = 2 |
| Bedtime | Time | Early or equal to 9 pm = 1; Later than 9 pm = 2 |
| Settling time (latency to falling asleep for the night) | Minute | <20 minutes = 1; ≥20 minutes = 2 |
| Nocturnal sleep duration (7 pm - 7 am) | Hour | Total sleep duration: |
| Daytime sleep duration (7 am - 7 pm) |  | 12-16 hours (6 months) / 11-14 hours (12 months) = 1; Other = 2 |
| Snoring | Never = 1; Occasionally = 2; Sometimes = 3; Almost always = 4; Only when sick or allergic = 5 | Never = 1; Other = 2 |
| Parent-reported sleep difficulties | No difficulty = 1; Slight difficulty = 2; Moderate difficulty = 3; Great difficulty = 4 | No difficulty = 1; Other = 2 |
| Average night wakings (10 pm - 6 am) | Times | <2 = 1; ≥2 = 2 |
| Sleep Regularity* | Irregular = 0; Regular = 1 | / |

* This item was not included in the LCA model.

Table S2. Domains assessed by the Strengths and Difficulties Questionnaire (SDQ), the Social Responsiveness Scale-Short Form (SRS-SF), and the Behavior Rating Inventory of Executive Function-Preschool Version (BRIEF-P)

|  | Subscale/index/composite score | Components included | Description |
| --- | --- | --- | --- |
| SDQ | Emotional Symptoms |  | Emotional symptoms, including anxiety, worries, low mood, fears, and somatic complaints. |
|  | Conduct Problems |  | Conduct-related difficulties, including oppositional, irritable, or aggressive behaviors. |
|  | Hyperactivity |  | Hyperactivity, impulsivity, restlessness, and difficulty sustaining attention. |
|  | Peer Relationship Problems |  | Difficulties in peer interaction, social acceptance, and age-appropriate peer relationships. |
|  | Prosocial Behavior |  | Prosocial behaviors, including empathy, helping, sharing, and consideration for others. Higher scores indicating better prosocial behavior. |
|  | Total Difficulties | Emotional Symptoms + Conduct Problems + Hyperactivity + Peer Relationship Problems | Overall emotional and behavioral problems. Higher scores indicate greater difficulties. |
| SRS-SF | Social Cognition |  | Difficulties in interpreting social cues and understanding social meaning. |
|  | Social Communication |  | Difficulties in reciprocal social communication and interaction with peers or others. |
|  | Autistic Mannerisms |  | Restricted interests, repetitive behaviors, inflexibility, and atypical behavioral patterns. |
|  | Total Score | Social Cognition + Social Communication + Autistic Mannerisms | Overall severity of social difficulties and autistic traits. Higher scores indicate greater difficulties. |
| BRIEF-P | Inhibit |  | Inhibitory control, impulse control, and ability to stop or modulate behavior. |
|  | Shift |  | Cognitive and behavioral flexibility, including the ability to shift between activities or situations. |
|  | Emotional Control |  | Ability to regulate and modulate emotional responses. |
|  | Working Memory |  | Ability to hold information in mind and sustain activity to complete a task. |
|  | Plan/Organize |  | Ability to anticipate, plan, organize, and carry out future-oriented problem-solving. |
|  | Inhibitory Self-Control Index (ISCI) | Inhibit + Emotional Control | Behavioral and emotional self-control through inhibitory regulation. |
|  | Flexibility Index (FI) | Shift + Emotional Control | Flexibility in actions, responses, emotions, and behavior. |
|  | Emergent Metacognition Index (EMI) | Working Memory + Plan/Organize | Emerging metacognitive abilities, including working memory, planning, and organization. |
|  | Global Executive Composite (GEC) | Inhibit + Shift + Emotional Control + Working Memory + Plan/Organize | Overall executive function. Higher scores indicate poorer executive function. |

Table S3. Internal Consistency of Child Neurodevelopmental and Behavioral Outcome Measures

|  | Subscale/index/composite score | Number of items | Cronbach’s α |
| --- | --- | --- | --- |
| SDQ | Emotional Symptoms | 5 | 0.50 |
|  | Conduct Problems | 5 | 0.38 |
|  | Hyperactivity | 5 | 0.77 |
|  | Peer Relationship Problems | 5 | 0.34 |
|  | Prosocial Behavior | 5 | 0.68 |
|  | Total difficulties | 20 | 0.72 |
| SRS-SF | Social Cognition | 5 | 0.35 |
|  | Social Communication | 8 | 0.60 |
|  | Autistic Mannerisms | 5 | 0.45 |
|  | Total Score | 18 | 0.70 |
| BRIEF-P | Inhibit | 16 | 0.85 |
|  | Shift | 10 | 0.80 |
|  | Emotional Control | 10 | 0.78 |
|  | Working Memory | 17 | 0.86 |
|  | Organize | 10 | 0.74 |
|  | ISCI | 26 | 0.88 |
|  | FI | 20 | 0.87 |
|  | EMI | 27 | 0.90 |
|  | GEC | 63 | 0.94 |

Table S4. Demographic Characteristics of Participants Included in and Excluded from Analyses

| Characteristic | Overall^1^ | Excluded^1^ | Included^1^ | p-value^2^ |
| --- | --- | --- | --- | --- |
|  | N = 1947 | N = 240 | N = 1707 |  |
| **Child sex** |  |  |  | >0.900 |
| *Boys* | 1021(52.47) | 126(52.72) | 895(52.43) |  |
| *Girls* | 925(47.53) | 113(47.28) | 812(47.57) |  |
| *Missing* | 1 | 1 | 0 |  |
| **Child age, mean (SD), years** | 4.49(0.26) | 4.44(0.24) | 4.50(0.27) | **<0.001** |
| *Missing* | 2 | 2 | 0 |  |
| **Breastfeeding Status at 6 Months** |  |  |  | 0.600 |
| *Not Breastfed* | 84(5.05) | 3(3.33) | 81(5.15) |  |
| *Breastfed* | 1578(94.95) | 87(96.67) | 1491(94.85) |  |
| *Missing* | 285 | 150 | 135 |  |
| **Breastfeeding Status at 12 Months** |  |  |  | 0.300 |
| *Never breastfed* | 45(2.54) | 2(1.68) | 43(2.60) |  |
| *Stopped Breastfeeding* | 1162(65.65) | 71(59.66) | 1091(66.08) |  |
| *Still Breastfeeding* | 563(31.81) | 46(38.66) | 517(31.31) |  |
| *Missing* | 177 | 121 | 56 |  |
| **Primary Caregiver's Education at 6 Months** |  |  |  | 0.700 |
| *Below High School* | 502(31.73) | 16(27.12) | 486(31.91) |  |
| *High School* | 425(26.86) | 18(30.51) | 407(26.72) |  |
| *Above High School* | 655(41.40) | 25(42.37) | 630(41.37) |  |
| *Missing* | 365 | 181 | 184 |  |
| **Primary Caregiver's Education at 12 Months** |  |  |  | 0.400 |
| *Below High School* | 719(44.44) | 21(36.84) | 698(44.71) |  |
| *High School* | 503(31.09) | 18(31.58) | 485(31.07) |  |
| *Above High School* | 396(24.47) | 18(31.58) | 378(24.22) |  |
| *Missing* | 329 | 183 | 146 |  |
|  |  |  |  |  |
| **Maternal Ethnicity** |  |  |  | 0.200 |
| *Han* | 1851(98.67) | 220(97.78) | 1631(98.79) |  |
| *Non-Han* | 25(1.33) | 5(2.22) | 20(1.21) |  |
| *Missing* | 71 | 15 | 56 |  |
| **Maternal age at delivery, mean (SD), years** | 28.79(3.70) | 29.06(3.73) | 28.76(3.70) | 0.200 |
| *Missing* | 73 | 15 | 58 |  |
| **Parity** |  |  |  | 0.069 |
| *0* | 1599(85.42) | 182(81.25) | 1417(85.98) |  |
| *≥1* | 273(14.58) | 42(18.75) | 231(14.02) |  |
| *Missing* | 75 | 16 | 59 |  |
| **Premature Birth** |  |  |  | 0.300 |
| *Yes* | 90(4.89) | 12(6.45) | 78(4.71) |  |
| *No* | 1751(95.11) | 174(93.55) | 1577(95.29) |  |
| *Missing* | 106 | 54 | 52 |  |
| **Maternal prenatal anxiety** |  |  |  | 0.300 |
| *Normal* | 1649(93.59) | 175(91.15) | 1474(93.89) |  |
| *Minimal* | 102(5.79) | 16(8.33) | 86(5.48) |  |
| *Moderate* | 11(0.62) | 1(0.52) | 10(0.64) |  |
| *Missing* | 185 | 48 | 137 |  |
| **Maternal education** |  |  |  | 0.500 |
| *Less than bachelor’s degree* | 650(34.65) | 71(31.56) | 579(35.07) |  |
| *Bachelor’s degree* | 992(52.88) | 122(54.22) | 870(52.70) |  |
| *Graduate degree* | 234(12.47) | 32(14.22) | 202(12.24) |  |
| *Missing* | 71 | 15 | 56 |  |
| **Self-Reported Family Economic Status** |  |  |  | 0.400 |
| *Adequate* | 334(18.34) | 36(16.90) | 298(18.53) |  |
| *Moderate* | 1283(70.46) | 158(74.18) | 1125(69.96) |  |
| *Tight* | 204(11.20) | 19(8.92) | 185(11.50) |  |
| *Missing* | 126 | 27 | 99 |  |

^1^Mean (SD) or n (%)

^2^Fisher's exact test; Welch Two Sample t-test

Table S5. Infant Sleep Characteristics at 6 and 12 Months of Age (N=1,707)

| Sleep Variable | 6-month old^1^ | 12-month old^1^ |
| --- | --- | --- |
| **Sleep location** |  |  |
| *Room-sharing with parents* | 715(47.19) | 552(34.85) |
| *Bed-sharing with parents* | 693(45.74) | 888(56.06) |
| *Crib in own room* | 31(2.05) | 31(1.96) |
| *Room-sharing with sibling* | 76(5.02) | 113(7.13) |
| *Missing* | 192 | 123 |
| **Preferred body position** |  |  |
| *Back* | 1046(68.46) | 608(38.46) |
| *Side* | 418(27.36) | 741(46.87) |
| *Stomach* | 64(4.19) | 232(14.67) |
| *Missing* | 179 | 126 |
| **Sleep initiation methods** |  |  |
| *In bed alone* | 167(10.94) | 227(14.46) |
| *Feeding to sleep* | 726(47.54) | 534(34.01) |
| *Rocking to sleep* | 100(6.55) | 84(5.35) |
| *Being held to sleep* | 377(24.69) | 343(21.85) |
| *In bed near parent* | 157(10.28) | 382(24.33) |
| *Missing* | 180 | 137 |
| **Bedtime** |  |  |
| *20-21:00* | 920(61.33) | 1118(71.21) |
| *<20:00* | 308(20.53) | 149(9.49) |
| *>21:00* | 272(18.13) | 303(19.30) |
| *Missing* | 207 | 137 |
| **Snoring** |  |  |
| *Never* | 897(58.44) | 846(53.27) |
| *Occasionally* | 376(24.50) | 661(41.62) |
| *Sometimes* | 218(14.20) | 41(2.58) |
| *Almost always* | 15(0.98) | 10(0.63) |
| *Only when sick or allergic* | 29(1.89) | 30(1.89) |
| *Missing* | 172 | 119 |
| **Parental Perception of Child Sleep Difficulty** |  |  |
| *No difficulty* | 1150(75.26) | 1192(75.30) |
| *Slight difficulty* | 263(17.21) | 290(18.32) |
| *Moderate difficulty* | 102(6.68) | 85(5.37) |
| *Great difficulty* | 13(0.85) | 16(1.01) |
| *Missing* | 179 | 124 |
| **Average night wakings, mean (SD)** | 1.47±1.09 | 1.17±1.01 |
| Missing | 267 | 125 |
| **Settling time, mean (SD), minutes** | 20.47±14.57 | 19.83±13.43 |
| Missing | 189 | 129 |
| **Total sleep duration, mean (SD), hours** | 13.03±1.42 | 12.62±1.10 |
| Missing | 177 | 130 |

^1^Mean (SD) or n (%)

Table S6. Model fit indices and class distribution for the 6-month latent class analysis (LCA) models

| Number of latent classes | AIC | BIC | G^2^ | X^2^ | Entropy | Class distribution, n (%) |
| --- | --- | --- | --- | --- | --- | --- |
| 1 | 13649.27 | 13696.09 | 823.65 | 1162.58 |  | 1: 1342 (100.0%) |
| 2 | 13359.64 | 13458.48 | 514.02 | 657.67 | 0.553 | 1: 886 (66.0%); 2: 456 (34.0%) |
| 3 | 13315.48 | 13466.33 | 449.85 | 560.65 | 0.591 | 1: 686 (51.1%); 2: 173 (12.9%); 3: 483 (36.0%) |
| 4 | 13298.70 | 13501.57 | 413.07 | 546.05 | 0.499 | 1: 193 (14.4%); 2: 304 (22.7%); 3: 666 (49.6%); 4: 179 (13.3%) |
| 5 | 13298.25 | 13553.14 | 392.62 | 544.71 | 0.588 | 1: 228 (17.0%); 2: 244 (18.2%); 3: 127 (9.5%); 4: 587 (43.7%); 5: 156 (11.6%) |
| 6 | 13296.32 | 13603.23 | 370.7 | 467.55 | 0.513 | 1: 121 (9.0%); 2: 169 (12.6%); 3: 114 (8.5%); 4: 186 (13.9%); 5: 242 (18.0%); 6: 510 (38.0%) |

Abbreviations: AIC, Akaike information criterion; BIC, Bayesian information criterion; G², likelihood-ratio chi-square statistic; X², Pearson chi-square statistic.

Table S7. Model fit indices and class distribution for the 12-month latent class analysis (LCA) models

| Number of latent classes | AIC | BIC | G^2^ | X^2^ | Entropy | Class distribution, n (%) |
| --- | --- | --- | --- | --- | --- | --- |
| 1 | 14794.52 | 14842.24 | 996.05 | 1552.16 |  | 1: 1483 (100.0%) |
| 2 | 14309.49 | 14410.22 | 491.02 | 641.07 | 0.661 | 1: 457 (30.8%); 2: 1026 (69.2%) |
| 3 | 14265.34 | 14419.09 | 426.86 | 508.39 | 0.522 | 1: 640 (43.2%); 2: 428 (28.9%); 3: 415 (28.0%) |
| 4 | 14260.61 | 14467.38 | 402.14 | 429.97 | 0.544 | 1: 312 (21.0%); 2: 86 (5.8%); 3: 699 (47.1%); 4: 386 (26.0%) |
| 5 | 14251.33 | 14511.12 | 372.85 | 402.55 | 0.614 | 1: 322 (21.7%); 2: 570 (38.4%); 3: 164 (11.1%); 4: 377 (25.4%); 5: 50 (3.4%) |
| 6 | 14252.95 | 14565.76 | 354.48 | 392.72 | 0.612 | 1: 161 (10.9%); 2: 569 (38.4%); 3: 386 (26.0%); 4: 50 (3.4%); 5: 114 (7.7%); 6: 203 (13.7%) |

Abbreviations: AIC, Akaike information criterion; BIC, Bayesian information criterion; G², likelihood-ratio chi-square statistic; X², Pearson chi-square statistic.

Table S8. Associations between Sleep Patterns at 6 and 12 Months and Preschool Neurodevelopmental and Behavioral Outcomes, with Additional Adjustment for Preterm Birth

|  |  | Sleep patterns at 6 months | | Sleep patterns at 12 months | |
| --- | --- | --- | --- | --- | --- |
|  |  | Good (N=886) | Poor (N=456) | Good (N=1026) | Poor (N=457) |
|  |  |  | β (95% CI) | β (95% CI) | β (95% CI) |
| **SDQ** | Emotional Symptoms | 0 (Ref) | 0.14 (-0.05, 0.32) | 0 (Ref) | **0.27 (0.08, 0.46)** |
|  | Conduct Problems | 0 (Ref) | 0.09 (-0.05, 0.24) | 0 (Ref) | **0.26 (0.11, 0.41)** |
|  | Hyperactivity | 0 (Ref) | 0.21 (-0.06, 0.47) | 0 (Ref) | **0.29 (0.02, 0.56)** |
|  | Peer Relationship Problems | 0 (Ref) | 0.13 (-0.05, 0.31) | 0 (Ref) | 0.13 (-0.05, 0.31) |
|  | Prosocial Behavior | 0 (Ref) | -0.13 (-0.35, 0.09) | 0 (Ref) | **-0.39 (-0.61, -0.17)** |
|  | Total difficulties | 0 (Ref) | **0.56 (0.06, 1.07)** | 0 (Ref) | **0.95 (0.43, 1.47)** |
| **SRS-SF** | Social Cognition | 0 (Ref) | 0.22 (0.00, 0.44) | 0 (Ref) | **0.37 (0.14, 0.59)** |
|  | Social Communication | 0 (Ref) | 0.14 (-0.11, 0.39) | 0 (Ref) | 0.20 (-0.06, 0.46) |
|  | Autistic Mannerisms | 0 (Ref) | 0.12 (-0.04, 0.29) | 0 (Ref) | **0.19 (0.02, 0.36)** |
|  | Total Score | 0 (Ref) | 0.49 (0.00, 0.99) | 0 (Ref) | **0.75 (0.25, 1.25)** |
| **BRIEF-P** | Inhibit | 0 (Ref) | 0.56 (-0.33, 1.45) | 0 (Ref) | **1.22 (0.34, 2.11)** |
|  | Shift | 0 (Ref) | 0.23 (-0.65, 1.10) | 0 (Ref) | **1.23 (0.38, 2.08)** |
|  | Emotional Control | 0 (Ref) | 0.32 (-0.64, 1.28) | 0 (Ref) | **1.51 (0.57, 2.46)** |
|  | Working Memory | 0 (Ref) | 0.21 (-1.00, 1.41) | 0 (Ref) | **1.50 (0.29, 2.71)** |
|  | Organize | 0 (Ref) | 0.22 (-0.82, 1.25) | 0 (Ref) | **1.41 (0.37, 2.44)** |
|  | ISCI | 0 (Ref) | 0.56 (-0.36, 1.49) | 0 (Ref) | **1.49 (0.57, 2.40)** |
|  | FI | 0 (Ref) | 0.34 (-0.60, 1.28) | 0 (Ref) | **1.59 (0.68, 2.51)** |
|  | EMI | 0 (Ref) | 0.24 (-0.92, 1.40) | 0 (Ref) | **1.57 (0.41, 2.73)** |
|  | GEC | 0 (Ref) | 0.41 (-0.66, 1.47) | 0 (Ref) | **1.76 (0.70, 2.82)** |

Adjusted for Child sex, Child age, Maternal ethnicity, Maternal age at delivery, Parity, Maternal education, Maternal prenatal anxiety, Self-reported family economic status, Recruitment hospital, Breastfeeding status at 6/12 months, Primary caregiver's education at 6 and 12 months, and Preterm Birth.

The BRIEF-P did not adjust for Child sex and age because we used standardized T-scores.

Abbreviations: β: coefficients; CI: confidence interval; SDQ, Strengths and Difficulties Questionnaire; SRS-SF, Social Responsiveness Scale–Short Form; BRIEF-P, Behavior Rating Inventory of Executive Function–Preschool Version; ISCI, Inhibitory Self-Control Index; FI, Flexibility Index; EMI, Emergent Metacognition Index; GEC, Global Executive Composite.

Table S9. Associations between Changes in Infant Sleep Patterns from 6 to 12 Months and Preschool Neurodevelopmental and Behavioral Outcomes, with Additional Adjustment for Preterm Birth (N = 1,115)

|  |  | Consistently good (N=575) | Improved (N=186) | Deteriorated (N=161) | Consistently poor (N=193) |  |
| --- | --- | --- | --- | --- | --- | --- |
|  |  |  | β (95% CI) | β (95% CI) | β (95% CI) | *P* for trend |
| **SDQ** | Emotional Symptoms | 0 (Ref) | 0.18 (-0.09, 0.45) | 0.15 (-0.14, 0.45) | **0.30 (0.02, 0.57)** | **0.042** |
|  | Conduct Problems | 0 (Ref) | -0.02 (-0.23, 0.18) | 0.16 (-0.06, 0.38) | **0.24 (0.03, 0.45)** | **0.014** |
|  | Hyperactivity | 0 (Ref) | 0.25 (-0.14, 0.64) | 0.21 (-0.21, 0.62) | 0.38 (-0.02, 0.78) | 0.069 |
|  | Peer Relationship Problems | 0 (Ref) | 0.09 (-0.18, 0.35) | 0.07 (-0.21, 0.36) | 0.27 (-0.01, 0.54) | 0.078 |
|  | Prosocial Behavior | 0 (Ref) | -0.19 (-0.50, 0.12) | **-0.44 (-0.78, -0.10)** | **-0.56 (-0.89, -0.24)** | **<0.001** |
|  | Total difficulties | 0 (Ref) | 0.49 (-0.25, 1.22) | 0.59 (-0.20, 1.39) | **1.19 (0.43, 1.95)** | **0.003** |
| **SRS-SF** | Social Cognition | 0 (Ref) | 0.18 (-0.14, 0.50) | 0.27 (-0.07, 0.62) | **0.54 (0.21, 0.88)** | **0.001** |
|  | Social Communication | 0 (Ref) | 0.16 (-0.21, 0.52) | 0.01 (-0.38, 0.41) | 0.31 (-0.07, 0.69) | 0.176 |
|  | Autistic Mannerisms | 0 (Ref) | 0.12 (-0.12, 0.36) | 0.01 (-0.25, 0.27) | **0.31 (0.06, 0.56)** | **0.037** |
|  | Total Score | 0 (Ref) | 0.46 (-0.26, 1.18) | 0.30 (-0.47, 1.07) | **1.18 (0.44, 1.92)** | **0.004** |
| **BRIEF-P** | Inhibit | 0 (Ref) | 0.65 (-0.61, 1.91) | 0.82 (-0.54, 2.18) | **1.99 (0.69, 3.28)** | **0.004** |
|  | Shift | 0 (Ref) | 0.17 (-1.07, 1.40) | 1.14 (-0.19, 2.46) | **1.38 (0.12, 2.65)** | **0.016** |
|  | Emotional Control | 0 (Ref) | 0.41 (-0.93, 1.76) | **1.55 (0.10, 3.00)** | **1.76 (0.38, 3.14)** | **0.005** |
|  | Working Memory | 0 (Ref) | 0.09 (-1.66, 1.83) | 1.20 (-0.68, 3.08) | 1.66 (-0.13, 3.46) | **0.045** |
|  | Organize | 0 (Ref) | 0.31 (-1.17, 1.80) | **1.90 (0.30, 3.49)** | 1.42 (-0.11, 2.94) | **0.020** |
|  | ISCI | 0 (Ref) | 0.65 (-0.65, 1.95) | 1.21 (-0.19, 2.61) | **2.13 (0.80, 3.47)** | **0.001** |
|  | FI | 0 (Ref) | 0.37 (-0.94, 1.67) | **1.56 (0.15, 2.96)** | **1.80 (0.46, 3.14)** | **0.003** |
|  | EMI | 0 (Ref) | 0.18 (-1.49, 1.86) | 1.57 (-0.23, 3.37) | 1.71 (-0.01, 3.43) | **0.025** |
|  | GEC | 0 (Ref) | 0.45 (-1.06, 1.96) | 1.62 (-0.01, 3.25) | **2.14 (0.59, 3.70)** | **0.003** |

Adjusted for Child sex, Child age, Maternal ethnicity, Maternal age at delivery, Parity, Maternal education, Maternal prenatal anxiety, Self-reported family economic status, Recruitment hospital, Breastfeeding status at 6 months, Primary caregiver's education at 6 and 12 months, and Preterm Birth.

The BRIEF-P did not adjust for Child sex and age because we used standardized T-scores.

Abbreviations: β: coefficients; CI: confidence interval; SDQ, Strengths and Difficulties Questionnaire; SRS-SF, Social Responsiveness Scale–Short Form; BRIEF-P, Behavior Rating Inventory of Executive Function–Preschool Version; ISCI, Inhibitory Self-Control Index; FI, Flexibility Index; EMI, Emergent Metacognition Index; GEC, Global Executive Composite.

Table S10. Associations between Sleep Patterns at 6 and 12 Months and Preschool Neurodevelopmental and Behavioral Outcomes, with Additional Adjustment for Stress Disorders

|  |  | Sleep patterns at 6 months | | Sleep patterns at 12 months | |
| --- | --- | --- | --- | --- | --- |
|  |  | Good (N=886) | Poor (N=456) | Good (N=1026) | Poor (N=457) |
|  |  |  | β (95% CI) | β (95% CI) | β (95% CI) |
| **SDQ** | Emotional Symptoms | 0 (Ref) | 0.14 (-0.05, 0.32) | 0 (Ref) | **0.25 (0.06, 0.44)** |
|  | Conduct Problems | 0 (Ref) | 0.09 (-0.05, 0.24) | 0 (Ref) | **0.25 (0.11, 0.40)** |
|  | Hyperactivity | 0 (Ref) | 0.21 (-0.06, 0.48) | 0 (Ref) | **0.28 (0.01, 0.55)** |
|  | Peer Relationship Problems | 0 (Ref) | 0.13 (-0.05, 0.31) | 0 (Ref) | 0.12 (-0.06, 0.31) |
|  | Prosocial Behavior | 0 (Ref) | -0.14 (-0.36, 0.08) | 0 (Ref) | **-0.40 (-0.62, -0.18)** |
|  | Total difficulties | 0 (Ref) | **0.58 (0.07, 1.08)** | 0 (Ref) | **0.91 (0.39, 1.43)** |
| **SRS-SF** | Social Cognition | 0 (Ref) | 0.22 (-0.01, 0.44) | 0 (Ref) | **0.37 (0.15, 0.59)** |
|  | Social Communication | 0 (Ref) | 0.15 (-0.11, 0.40) | 0 (Ref) | 0.18 (-0.08, 0.45) |
|  | Autistic Mannerisms | 0 (Ref) | 0.13 (-0.04, 0.30) | 0 (Ref) | **0.18 (0.01, 0.35)** |
|  | Total Score | 0 (Ref) | **0.50 (0.01, 1.00)** | 0 (Ref) | **0.73 (0.24, 1.23)** |
| **BRIEF-P** | Inhibit | 0 (Ref) | 0.63 (-0.25, 1.51) | 0 (Ref) | **1.16 (0.28, 2.03)** |
|  | Shift | 0 (Ref) | 0.25 (-0.63, 1.12) | 0 (Ref) | **1.19 (0.34, 2.04)** |
|  | Emotional Control | 0 (Ref) | 0.37 (-0.58, 1.33) | 0 (Ref) | **1.43 (0.50, 2.37)** |
|  | Working Memory | 0 (Ref) | 0.23 (-0.97, 1.43) | 0 (Ref) | **1.44 (0.24, 2.64)** |
|  | Organize | 0 (Ref) | 0.26 (-0.77, 1.29) | 0 (Ref) | **1.36 (0.32, 2.39)** |
|  | ISCI | 0 (Ref) | 0.63 (-0.28, 1.55) | 0 (Ref) | **1.41 (0.50, 2.31)** |
|  | FI | 0 (Ref) | 0.38 (-0.55, 1.31) | 0 (Ref) | **1.52 (0.62, 2.43)** |
|  | EMI | 0 (Ref) | 0.28 (-0.88, 1.43) | 0 (Ref) | **1.51 (0.36, 2.67)** |
|  | GEC | 0 (Ref) | 0.46 (-0.60, 1.52) | 0 (Ref) | **1.69 (0.65, 2.74)** |

Adjusted for Child sex, Child age, Maternal ethnicity, Maternal age at delivery, Parity, Maternal education, Maternal prenatal anxiety, Self-reported family economic status, Recruitment hospital, Breastfeeding status at 6/12 months, and Primary caregiver's education at 6/12 months, CSDC total score.

The BRIEF-P did not adjust for Child sex and age because we used standardized T-scores.

Abbreviations: β: coefficients; CI: confidence interval; SDQ, Strengths and Difficulties Questionnaire; SRS-SF, Social Responsiveness Scale–Short Form; BRIEF-P, Behavior Rating Inventory of Executive Function–Preschool Version; ISCI, Inhibitory Self-Control Index; FI, Flexibility Index; EMI, Emergent Metacognition Index; GEC, Global Executive Composite.

Table S11. Associations between Changes in Infant Sleep Patterns from 6 to 12 Months and Preschool Neurodevelopmental and Behavioral Outcomes, with Additional Adjustment for Stress Disorders (N = 1,115)

|  |  | Consistently good (N=575) | Improved (N=186) | Deteriorated (N=161) | Consistently poor (N=193) |  |
| --- | --- | --- | --- | --- | --- | --- |
|  |  |  | β (95% CI) | β (95% CI) | β (95% CI) | *P* for trend |
| **SDQ** | Emotional Symptoms | 0 (Ref) | 0.16 (-0.11, 0.43) | 0.13 (-0.16, 0.42) | **0.29 (0.01, 0.57)** | 0.050 |
|  | Conduct Problems | 0 (Ref) | -0.03 (-0.23, 0.18) | 0.15 (-0.07, 0.37) | **0.24 (0.03, 0.45)** | **0.015** |
|  | Hyperactivity | 0 (Ref) | 0.25 (-0.14, 0.64) | 0.20 (-0.22, 0.62) | 0.39 (-0.01, 0.79) | 0.068 |
|  | Peer Relationship Problems | 0 (Ref) | 0.08 (-0.18, 0.35) | 0.06 (-0.22, 0.35) | 0.27 (-0.01, 0.54) | 0.079 |
|  | Prosocial Behavior | 0 (Ref) | -0.19 (-0.51, 0.12) | **-0.44 (-0.78, -0.10)** | **-0.57 (-0.89, -0.25)** | **<0.001** |
|  | Total difficulties | 0 (Ref) | 0.47 (-0.27, 1.20) | 0.54 (-0.25, 1.33) | **1.19 (0.43, 1.94)** | **0.003** |
| **SRS-SF** | Social Cognition | 0 (Ref) | 0.18 (-0.14, 0.51) | 0.28 (-0.07, 0.62) | **0.55 (0.22, 0.88)** | **0.001** |
|  | Social Communication | 0 (Ref) | 0.15 (-0.22, 0.52) | -0.01 (-0.41, 0.38) | 0.32 (-0.06, 0.70) | 0.179 |
|  | Autistic Mannerisms | 0 (Ref) | 0.12 (-0.12, 0.36) | 0.01 (-0.25, 0.27) | **0.32 (0.07, 0.57)** | **0.035** |
|  | Total Score | 0 (Ref) | 0.46 (-0.26, 1.18) | 0.28 (-0.50, 1.05) | **1.19 (0.46, 1.93)** | **0.004** |
| **BRIEF-P** | Inhibit | 0 (Ref) | 0.62 (-0.63, 1.87) | 0.67 (-0.68, 2.02) | **2.01 (0.73, 3.29)** | **0.004** |
|  | Shift | 0 (Ref) | 0.16 (-1.08, 1.39) | 1.08 (-0.24, 2.41) | **1.39 (0.12, 2.65)** | **0.017** |
|  | Emotional Control | 0 (Ref) | 0.36 (-0.97, 1.70) | 1.40 (-0.04, 2.84) | **1.76 (0.38, 3.13)** | **0.006** |
|  | Working Memory | 0 (Ref) | 0.03 (-1.71, 1.77) | 1.05 (-0.82, 2.93) | 1.65 (-0.13, 3.43) | 0.051 |
|  | Organize | 0 (Ref) | 0.28 (-1.20, 1.76) | **1.79 (0.19, 3.38)** | 1.42 (-0.10, 2.94) | **0.022** |
|  | ISCI | 0 (Ref) | 0.61 (-0.68, 1.89) | 1.04 (-0.35, 2.43) | **2.15 (0.83, 3.47)** | **0.001** |
|  | FI | 0 (Ref) | 0.33 (-0.97, 1.63) | **1.45 (0.05, 2.85)** | **1.80 (0.46, 3.14)** | **0.003** |
|  | EMI | 0 (Ref) | 0.13 (-1.53, 1.80) | 1.43 (-0.36, 3.23) | 1.70 (-0.01, 3.41) | **0.027** |
|  | GEC | 0 (Ref) | 0.41 (-1.10, 1.91) | 1.47 (-0.15, 3.10) | **2.16 (0.61, 3.70)** | **0.003** |

Adjusted for Child sex, Child age, Maternal ethnicity, Maternal age at delivery, Parity, Maternal education, Maternal prenatal anxiety, Self-reported family economic status, Recruitment hospital, Breastfeeding status at 6 months, and Primary caregiver's education at 6 and 12 months, CSDC total score.

The BRIEF-P did not adjust for Child sex and age because we used standardized T-scores.

Abbreviations: β: coefficients; CI: confidence interval; SDQ, Strengths and Difficulties Questionnaire; SRS-SF, Social Responsiveness Scale–Short Form; BRIEF-P, Behavior Rating Inventory of Executive Function–Preschool Version; ISCI, Inhibitory Self-Control Index; FI, Flexibility Index; EMI, Emergent Metacognition Index; GEC, Global Executive Composite.

Table S12. Associations between Sleep Patterns at 6 and 12 Months and Preschool Neurodevelopmental and Behavioral Outcomes, with Additional Adjustment for Accident Occurrence

|  |  | Sleep patterns at 6 months | | Sleep patterns at 12 months | |
| --- | --- | --- | --- | --- | --- |
|  |  | Good (N=886) | Poor (N=456) | Good (N=1026) | Poor (N=457) |
|  |  |  | β (95% CI) | β (95% CI) | β (95% CI) |
| **SDQ** | Emotional Symptoms | 0 (Ref) | 0.13 (-0.05, 0.32) | 0 (Ref) | **0.26 (0.07, 0.45)** |
|  | Conduct Problems | 0 (Ref) | 0.09 (-0.05, 0.23) | 0 (Ref) | **0.26 (0.11, 0.41)** |
|  | Hyperactivity | 0 (Ref) | 0.21 (-0.06, 0.48) | 0 (Ref) | **0.28 (0.01, 0.55)** |
|  | Peer Relationship Problems | 0 (Ref) | 0.13 (-0.06, 0.31) | 0 (Ref) | 0.13 (-0.06, 0.31) |
|  | Prosocial Behavior | 0 (Ref) | -0.14 (-0.36, 0.08) | 0 (Ref) | **-0.39 (-0.61, -0.17)** |
|  | Total difficulties | 0 (Ref) | **0.56 (0.05, 1.06)** | 0 (Ref) | **0.92 (0.40, 1.44)** |
| **SRS-SF** | Social Cognition | 0 (Ref) | 0.22 (-0.01, 0.44) | 0 (Ref) | **0.36 (0.14, 0.58)** |
|  | Social Communication | 0 (Ref) | 0.13 (-0.12, 0.38) | 0 (Ref) | 0.18 (-0.08, 0.44) |
|  | Autistic Mannerisms | 0 (Ref) | 0.12 (-0.05, 0.29) | 0 (Ref) | **0.18 (0.01, 0.35)** |
|  | Total Score | 0 (Ref) | 0.48 (-0.01, 0.97) | 0 (Ref) | **0.72 (0.22, 1.22)** |
| **BRIEF-P** | Inhibit | 0 (Ref) | 0.51 (-0.36, 1.39) | 0 (Ref) | **1.13 (0.26, 2.00)** |
|  | Shift | 0 (Ref) | 0.20 (-0.68, 1.07) | 0 (Ref) | **1.17 (0.32, 2.02)** |
|  | Emotional Control | 0 (Ref) | 0.30 (-0.66, 1.25) | 0 (Ref) | **1.45 (0.51, 2.39)** |
|  | Working Memory | 0 (Ref) | 0.14 (-1.06, 1.33) | 0 (Ref) | **1.40 (0.20, 2.60)** |
|  | Organize | 0 (Ref) | 0.17 (-0.85, 1.20) | 0 (Ref) | **1.30 (0.28, 2.32)** |
|  | ISCI | 0 (Ref) | 0.52 (-0.39, 1.44) | 0 (Ref) | **1.39 (0.49, 2.30)** |
|  | FI | 0 (Ref) | 0.31 (-0.62, 1.25) | 0 (Ref) | **1.52 (0.61, 2.43)** |
|  | EMI | 0 (Ref) | 0.18 (-0.97, 1.33) | 0 (Ref) | **1.47 (0.32, 2.62)** |
|  | GEC | 0 (Ref) | 0.35 (-0.70, 1.41) | 0 (Ref) | **1.66 (0.62, 2.71)** |

Adjusted for Child sex, Child age, Maternal ethnicity, Maternal age at delivery, Parity, Maternal education, Maternal prenatal anxiety, Self-reported family economic status, Recruitment hospital, Breastfeeding status at 6/12 months, and Primary caregiver's education at 6/12 months, Accident occurrence.

The BRIEF-P did not adjust for Child sex and age because we used standardized T-scores.

Abbreviations: β: coefficients; CI: confidence interval; SDQ, Strengths and Difficulties Questionnaire; SRS-SF, Social Responsiveness Scale–Short Form; BRIEF-P, Behavior Rating Inventory of Executive Function–Preschool Version; ISCI, Inhibitory Self-Control Index; FI, Flexibility Index; EMI, Emergent Metacognition Index; GEC, Global Executive Composite.

Table S13. Associations between Changes in Infant Sleep Patterns from 6 to 12 Months and Preschool Neurodevelopmental and Behavioral Outcomes, with Additional Adjustment for Accident Occurrence (N = 1,115)

|  |  | Consistently good (N=575) | Improved (N=186) | Deteriorated (N=161) | Consistently poor (N=193) |  |
| --- | --- | --- | --- | --- | --- | --- |
|  |  |  | β (95% CI) | β (95% CI) | β (95% CI) | *P* for trend |
| **SDQ** | Emotional Symptoms | 0 (Ref) | 0.16 (-0.11, 0.43) | 0.14 (-0.15, 0.43) | **0.28 (0.01, 0.56)** | 0.052 |
|  | Conduct Problems | 0 (Ref) | -0.02 (-0.23, 0.18) | 0.16 (-0.06, 0.38) | **0.24 (0.03, 0.45)** | **0.015** |
|  | Hyperactivity | 0 (Ref) | 0.24 (-0.15, 0.62) | 0.19 (-0.23, 0.60) | 0.37 (-0.03, 0.77) | 0.079 |
|  | Peer Relationship Problems | 0 (Ref) | 0.08 (-0.18, 0.34) | 0.06 (-0.22, 0.35) | 0.26 (-0.01, 0.53) | 0.086 |
|  | Prosocial Behavior | 0 (Ref) | -0.18 (-0.50, 0.13) | **-0.43 (-0.76, -0.09)** | **-0.56 (-0.88, -0.24)** | **<0.001** |
|  | Total difficulties | 0 (Ref) | 0.46 (-0.28, 1.19) | 0.55 (-0.24, 1.34) | **1.16 (0.40, 1.92)** | **0.003** |
| **SRS-SF** | Social Cognition | 0 (Ref) | 0.18 (-0.15, 0.50) | 0.26 (-0.08, 0.61) | **0.54 (0.21, 0.87)** | **0.001** |
|  | Social Communication | 0 (Ref) | 0.13 (-0.23, 0.50) | -0.03 (-0.42, 0.37) | 0.29 (-0.09, 0.67) | 0.229 |
|  | Autistic Mannerisms | 0 (Ref) | 0.11 (-0.13, 0.36) | 0.01 (-0.25, 0.27) | **0.31 (0.06, 0.56)** | **0.040** |
|  | Total Score | 0 (Ref) | 0.44 (-0.28, 1.15) | 0.25 (-0.52, 1.01) | **1.15 (0.42, 1.88)** | **0.006** |
| **BRIEF-P** | Inhibit | 0 (Ref) | 0.51 (-0.73, 1.75) | 0.64 (-0.69, 1.98) | **1.86 (0.59, 3.12)** | **0.006** |
|  | Shift | 0 (Ref) | 0.10 (-1.13, 1.33) | 1.05 (-0.27, 2.37) | **1.32 (0.06, 2.58)** | **0.022** |
|  | Emotional Control | 0 (Ref) | 0.35 (-1.00, 1.69) | **1.47 (0.03, 2.92)** | **1.70 (0.32, 3.08)** | **0.006** |
|  | Working Memory | 0 (Ref) | -0.07 (-1.80, 1.66) | 1.03 (-0.83, 2.89) | 1.51 (-0.27, 3.28) | 0.068 |
|  | Organize | 0 (Ref) | 0.17 (-1.30, 1.64) | **1.73 (0.15, 3.31)** | 1.28 (-0.22, 2.79) | **0.032** |
|  | ISCI | 0 (Ref) | 0.52 (-0.76, 1.80) | 1.06 (-0.32, 2.44) | **2.02 (0.70, 3.34)** | **0.002** |
|  | FI | 0 (Ref) | 0.29 (-1.01, 1.59) | **1.46 (0.06, 2.86)** | **1.73 (0.39, 3.07)** | **0.004** |
|  | EMI | 0 (Ref) | 0.03 (-1.63, 1.68) | 1.40 (-0.39, 3.19) | 1.56 (-0.15, 3.26) | **0.038** |
|  | GEC | 0 (Ref) | 0.30 (-1.19, 1.79) | 1.46 (-0.15, 3.07) | **2.01 (0.48, 3.55)** | **0.005** |

Adjusted for Child sex, Child age, Maternal ethnicity, Maternal age at delivery, Parity, Maternal education, Maternal prenatal anxiety, Self-reported family economic status, Recruitment hospital, Breastfeeding status at 6 months, and Primary caregiver's education at 6 and 12 months, Accident occurrence.

The BRIEF-P did not adjust for Child sex and age because we used standardized T-scores.

Abbreviations: β: coefficients; CI: confidence interval; SDQ, Strengths and Difficulties Questionnaire; SRS-SF, Social Responsiveness Scale–Short Form; BRIEF-P, Behavior Rating Inventory of Executive Function–Preschool Version; ISCI, Inhibitory Self-Control Index; FI, Flexibility Index; EMI, Emergent Metacognition Index; GEC, Global Executive Composite.
